# Metachronous pancreatic metastases from renal cell carcinoma: is there a place of Active-Surveillance before deferred deliberately Molecular Target Agent?

**DOI:** 10.1186/s12957-016-0939-9

**Published:** 2016-08-23

**Authors:** Yassir Sbitti, Hassan Seddik, Adil Debbagh, Fahd Benani, Khaoula Slimani, Mohamed Mahi, Mohamed Tarchouli, Abdelmounaim Aitali, Abderrahmane Albouzidi, Hassan Errihani, Mohamed Ichou

**Affiliations:** 1 Department of Medical Oncology, University Military Hospital, Rabat, 10000 Morocco; 2 Department of Gastroenterology, University Military Hospital, Rabat, 10000 Morocco; 3 Department of Pathology, University Military Hospital of Instruction, Rabat, 10000 Morocco; 4 Department of Surgery, University Military Hospital of Instruction, Rabat, 10000 Morocco; 5 Department of Radiology, University Military Hospital of Instruction, Rabat, 10000 Morocco; 6 Department of Medical Oncology, National Institute of Oncology, Rabat, 10000 Morocco; 7 Department Medical Oncology, Teaching University Military Hospital and Faculty of Medicine and Pharmacy of Fes, Hay Ryad, Rabat, 10000 Morocco

**Keywords:** Renal cell carcinoma, Pancreatic metastases, Active surveillance, Molecular target therapy

## Abstract

**Background:**

Metastatic renal cell cancer is a heterogeneous disease due to its diverse morphological features, the prognostic categories based on clinical criteria. Sometimes indolent course without any significant symptoms can be differentiated before the introduction of novel targeted agents. This observation led to interest in a strategy of deferring systemic therapy in the era of effective systemic therapies.

**Case presentation:**

We report of a 78-year-old Moroccan man with pancreatic metastasis from renal cell carcinoma which occurred 14 years from right nephrectomy. Indolent disease based on body computed tomography imaging with 4 years follow-up was recognized. Active surveillance with deferred antiangiogenic multikinase inhibitor at disease progression was proposed. Nowadays, the patient is under oncological follow-up, he is in a good state of health, and he is disease-free for 48 months from the diagnosis of the tumor and for 20 months from the start of the treatment with Sunitinib

**Conclusions:**

Active surveillance before target therapy may be a suitable approach to ensure long progression-free survival with minimal side-effects and better quality of life in asymptomatic, low-volume, metastatic disease. Further prospective studies with biomarker validation are required to define the patients most likely to benefit from this approach.

## Background

Pancreatic Metastases from Renal Cell Carcinoma (RCC) are rare with less than 2 % reported in autopsy series [1, 2]. RCC usually leads to a solitary pancreatic metastasis, whereas multiple pancreatic metastases are uncommon [3]. Pancreatic metastases of RCC were rarely clinically or biologically symptomatic. Surgical treatment for pancreatic metastases from RCC has been reported in recent years to improve survival [4–7]. However, the opportunity for surgical exploration is limited. Surgical resection of the pancreas is associated with substantial morbidity while target therapy is recommended for non-surgical pancreatic lesion. Some data suggest an effect of antiangiogenic molecules on these hyper vascularized lesions for patients who are not eligible for surgery [8–10]. Several molecular target therapy for metastatic Renal Cell Carcinoma exist and goal of Treatments is still palliative. Moreover, all available target agents have considerable side’s effects that could compromise quality of life and cause economic burden to patient and society. However, renal cell carcinoma with metachronous pancreatic metastases represent a heterogeneous disease and sometimes disseminated disease presents with an indolent course without any symptom. Warrant careful surveillance before active systemic therapy may be a suitable approach to ensure survival with better quality of life. Here, we report a case of asymptomatic unresectable metastatic Renal Cell Carcinoma to pancreas managed by active surveillance and deferred Sunitinib.

## Case presentation

**Fig. 1:**
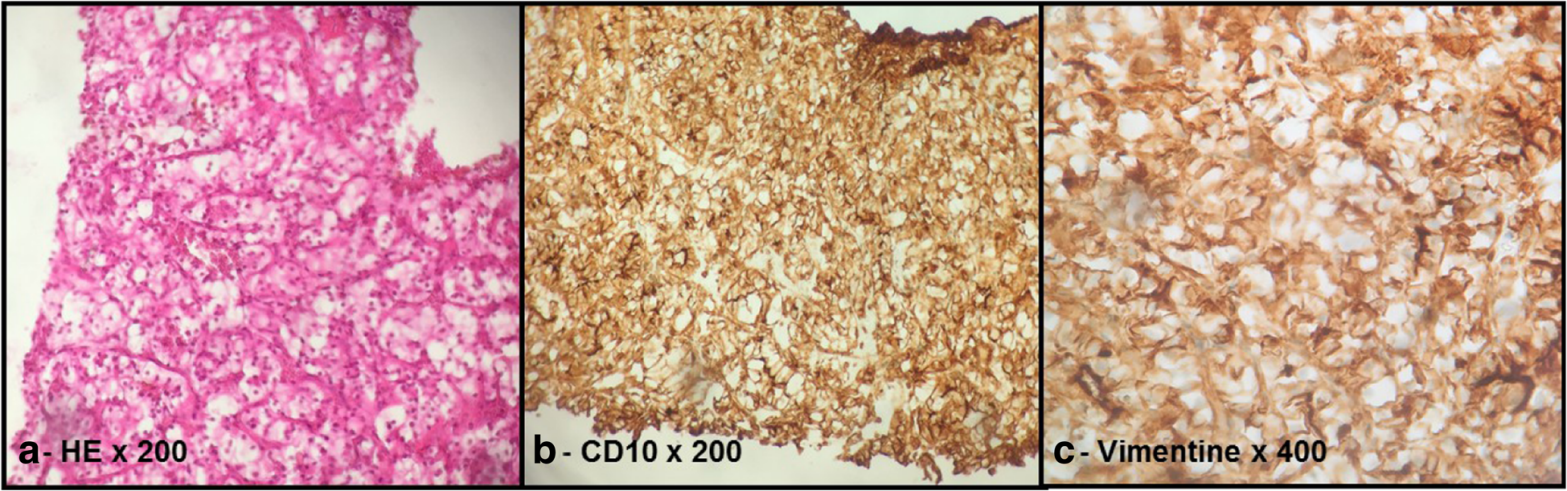
Histopathological findings of pancreatic metastases from renal cell cancer. **a** Magnification of head tumor with hematoxylin and eosin (H.E.) stain ×200. **b** and **c** Immunochemical study was performed. The tumor cells were strongly positive for CD10, EMA, and vimentin, which were consistent with metastatic renal cell carcinoma to pancreas

**Fig. 2:**
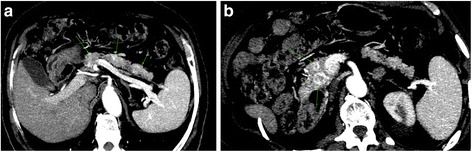
CT findings of the pancreatic metastases. **a** Body lesion with central necrosis and enhanced rim. **b** Homogenous hyper vascular head lesion

A 78-year-old man had undergone a right radical nephrectomy for renal cell carcinoma 14 years previously. The renal tumor measured 32 × 31 mm and was found in medium. The tumor was classified as grade 2 in the Fuhrman nuclear grading system (large clear granular cells). No capsular involvement was seen, and the ureter and hilar lymph nodes were tumor-free. Staging investigations, including a computed tomography (CT) scan, revealed no obvious metastasis. A bone scan was not carried out. The tumor was staged as T1, N0, and M0. He underwent a biannual medical examination and monitoring with regular CT scans. He remained well and asymptomatic until May 2010. When on surveillance CT scan, the patient was found to have a multiple enhancing lesion in the head (40 × 50 mm) body (35 × 42 mm) and tail (30 × 3 5 mm) of the pancreas. Due to his medical history of a right nephrectomy 16 years ago owing to renal cell carcinoma, a CT scan of the brain and the thorax was performed, which was negative for metastases. To asses diagnosis, a CT scan-guided biopsy was performed. Pancreatic biopsy showed neoplastic cells present singly and in loosely cohesive clusters. The cells were round to polygonal with clear cytoplasm. The nuclei were enlarged, round to oval. Mitotic index was low activity. Immunostaining was performed. The tumor cells were strongly positive for CD10 antibody, epithelial membrane antigen, and vimentin, which were consistent with metastatic renal cell carcinoma to pancreas (Fig. 1). In view of metastatic RCC with multifocal disease in pancreas, surgery was not accepted by the patient. He was therefore referred for systemic treatments. We decided to start a watchful waiting, considering that the disease was asymptomatic with low mitotic index. Heng risk was favorable. On active surveillance (AS), we perform a contrast-enhanced CT scan of the thorax, abdomen, and pelvis every 4 months. In February 2014, abdominal CT scan demonstrated an increase in the size of pancreatic lesions. Baseline cardiac evaluation was normal. So, the patient started medical treatment with sunitinib, 50 mg/day 6-week cycles of sunitinib, 4 weeks on and 2 weeks off. Evaluation of the tumor response was done according to response evaluation criteria in solid tumors by spiral CT scan, after three cycles of sunitinib observe a partial response (30 % reduction in size and 50 % density of pancreatic lesions) (Fig. 2). Due to the onset of grade III skin and mucosal toxicity and gastrointestinal toxicity, adverse events were managed through supportive care and dose interruption. Sunitinib rechallenge dosing schema was changed to 37.5 mg/day 2/3 schedule for other cycles. The patient tolerated this alternative Sunitinib schedule fairly well, but did develop grade II hypothyroidism managed by l thyroxin supplementation, and restaging CT scan after three cycles of this regimen continued to show stable disease consistent with partial response per response evaluation criteria in solid tumor. He is presently on the 12th cycle of oral sunitinib and is doing well with clinically asymptomatic status. Nowadays, the patient is under oncological follow-up, he was in a good state of health, and disease-control under active surveillance was 48 and 20 months from the start of sunitinib treatment.

## Conclusions

Renal Cell Carcinoma (RCC), melanoma, lung cancer, colorectal cancer, gynaecological and breast cancer are known to metastasize to the pancreas [11–15]. Solitary metastasis to the pancreas account from 2 to 5 % in clinical studies [15, 16]. They can occur many years after its removal especially if the tumor is well- differentiated. Pancreatic metastases from RCC have been recorded over the course of 6 months to 27 years following nephrectomy and 11 % of these metastases have been described in literature as occurring more than 10 years after the initial radical surgical procedure [16, 17]. In our case, the intervals between primary tumor resection and detection of metastasis to the pancreas were 16 years. The majority of pancreatic metastases renal cell carcinoma are asymptomatic and are often detected during follow-up investigations after surgery for a primary lesion or as an incidental finding on imaging studies done for an unrelated indication the tumour is often diagnosed as part of follow-up examinations as in our index case, whereas some exhibit jaundice or abdominal pain [18]. In general, preoperative diagnosis of pancreatic metastases begins with a suspicion based on the patient’s history [19, 20]. Multiples imaging modalities support the diagnosis. Muranaka et al. reviewed the CT findings of pancreatic metastases from 28 metastatic carcinomas and classified these into three types according to their configuration: (1) a single localized metastasis (50 to 73 %); (2) a diffuse enlargement with homogeneous attenuation of the pancreas (15 to 44 %), and (3) multifocal metastases (5 to 10 %) [21]. Metastases from RCC are usually hyper vascular and consequently display homogeneous contrast medium enhancement in the arterial phase of CT. Hyper enhancement of pancreatic metastases from RCC plays an important role in both the detection of tumor locations and the distinction of metastases from primary adenocarcinoma of the pancreas [22, 23]. In some cases, despite these radiological features, it can be difficult to distinguish a pancreatic RCC metastasis from a primary pancreatic ductal and lesions should be evaluated with biopsy [22, 23]. There are several factors affecting survival of patients with metastases of RCC: the site and number of foci, the performance status and the disease free interval of the patients [24, 25]. Pancreatic metastasectomy of RCC was reported to improve survival in selected patients [4–7]. The specific type of surgical resection will depend on the location of the tumor within the pancreas. However, in patients with multicentric pancreatic metastases, as in our case, the role of surgery is controversial, because a benefit of surgery on then cases remains questionable since randomized control trials have not been conducted, and total pancreatectomy is fairly uncommon and morbid [26]. Recently, introduction of Molecular Target Agents (MTAs) revolutionized the management of mRCC and we now have several first and second line treatments options [27–33]. While, not all patients with metastatic RCC behave similarly; some data suggest that there may be a specific subset of mRCC patients appears to have stable diseases without systemic therapy which means that mRCC consist of heterogeneous diseases with variable biology [34, 35]. Furthermore, chronic use of MTAs for mRCC entails chronic but moderate toxicities that could compromise quality of life and causes economic burden to patients and society [27–33]. Therefore, in light of slow disease progression, non-curative nature of current systemic therapy and the toxicity associated with MTAs, the application of customized treatment strategy according to disease aggressiveness seems a more rational approach than the immediate treatment for all patients regardless of disease status. In our patient, the average period observation was 48 months and the progression free survival (PFS) and overall survival (OS) for the subsequent sunitinib treatment were comparable with those observed in the phase III trial of sunitinib for mRCC [27]. Moreover in multiple pancreas metastases from RCC, especially asymptomatic patients with a long disease free interval (>5 years), without neurtrophilia or liver metastases, some date suggest that this subgroup of patients might possibly survive anyway without surgery and/or targeted agents [34–36]. Of the 260 new patient’s (pts) with mRCC, we identified 102 pts who were under a period of active surveillance as part of their management. The main reasons for AS were low volume asymptomatic disease in 71 pts (68.3 %) and comorbidities in 15 pts (14.4 %). Treatments prior to AS included: none 69 pts, surgery for oligometastatic disease 19 pts, radiotherapy 13 pts, and biologic treatment 4 pts. At the beginning of AS, the patients Motzer prognostic categories were: good in 35.6 %, intermediate in 58.7 % and poor in 5.8 % of pts. The number of metastatic sites was 1/2/≥ 3: 59 pts/26 pts/8 pts. The median time on AS was 11 months (95 % confidence interval CI: 8.8–13.1). With a median follow-up of 27.5 months, 56 pts had disease progression. Of these, 39 pts had new metastases but the MSKCC prognostic group deteriorated in only 5 pts. After disease progression, 42 pts started Tyrosine Kinase Inhibitor, with a median PFS of 10 months (95 % CI: 6.78–13.2). Sunitinib was the most common drug started (27 pts). The OS for the entire cohort (260 pts) was 32 months: 39 months for pts in AS compared with 17 months for those pts who weren't on AS (Hazard Ratio HR, 2.39; 95 % CI: 1.57–3.53; *P* < 0.0001) [35]. In summary, AS seems to be an acceptable approach to delay or avoid further target therapy and to improve quality of life. Candidates for AS include selected patients older than 70 years who have asymptomatic disease with slow growth documented on serial imaging as in our case. Treatments strategy of metastatic renal cell carcinoma is problematic, and, whenever possible, patients should be directed to approved and controlled clinical trials. To date none of the cancer guidelines recommends surveillance for mRCC. Our index case and some retrospective data suggests the consideration of AS based on expert decision may delay active treatment, probably without detriment to the OS [34–36]. The possibility of disease stabilization should be considered before initiation of any active treatment and when assessing the activity of any treatment in terms of time to progression [32–36]. Moreover, it is a reasonable approach for patients where due to comorbidities the risk of treatment could be greater than the benefit. A subset of mRCC pts can be safely observed for a period of time before starting systemic therapy. Multidisciplinary evaluation plays a crucial role in the management of patients with indolent course of the disease. Further observationnel studies might be needed.

## Abbreviations

AS, active surveillance; CT, computed tomography; mRCC, metastatic renal cell carcinoma; MTAs, molecular target agents; OS, overall survival; PFS, progression-free survival.
